# Phelan-McDermid syndrome: a classification system after 30 years of experience

**DOI:** 10.1186/s13023-022-02180-5

**Published:** 2022-01-29

**Authors:** Katy Phelan, Luigi Boccuto, Craig M. Powell, Tobias M. Boeckers, Conny van Ravenswaaij-Arts, R. Curtis Rogers, Carlo Sala, Chiara Verpelli, Audrey Thurm, William E. Bennett, Christopher J. Winrow, Sheldon R. Garrison, Roberto Toro, Thomas Bourgeron

**Affiliations:** 1 Genetics Laboratory, Florida Cancer Specialists, Fort Myers, FL 33916 USA; 2 School of Nursing, College of Behavioral, Social and Health Sciences, Clemson University, Clemson, SC 29634 USA; 3 Department of Neurobiology, University of Alabama at Birmingham, Marnix E. Heersink School of Medicine, Birmingham, AL 35233 USA; 4 Institute of Anatomy and Cell Biology, University of Ulm, Ulm, Germany; 5 Present Address: Medical Centre Groningen, Department of Genetics, University of Groningen, Groningen, Netherlands; 6 Greenwood Genetic Center – Greenville, Greenville, SC 29605 USA; 7 CNR Neuroscience Institute, University of Milan, Milan, Italy; 8 Neurodevelopmental and Behavioral Phenotyping Service, NIMH, Bethesda, MD 20892 USA; 9 Division of Pediatrics, Gastroenterology, Hepatology, and Nutrition, Department of Pediatrics, Indiana University School of Medicine, Indianapolis, IN 46202 USA; 10 Cyclerion Therapeutics, Cambridge, MA 02142 USA; 11 Rogers Behavioral Health, Oconomowoc, WI 53066 USA; 12 Human Genetics and Cognitive Functions, Institut Pasteur, UMR3571 CNRS, Université de Paris, 75015 Paris, France

**Keywords:** Phelan-McDermid syndrome, PMS, *SHANK3*, 22q13 deletion

## Abstract

Phelan-McDermid syndrome (PMS) was initially called the 22q13 deletion syndrome based on its etiology as a deletion of the distal long arm of chromosome 22. These included terminal and interstitial deletions, as well as other structural rearrangements. Later, pathogenetic variants and deletions of the *SHANK3* gene were found to result in a phenotype consistent with PMS. The association between *SHANK3* and PMS led investigators to consider disruption/deletion of *SHANK3* to be a prerequisite for diagnosing PMS. This narrow definition of PMS based on the involvement of *SHANK3* has the adverse effect of causing patients with interstitial deletions of chromosome 22 to “lose” their diagnosis. It also results in underreporting of individuals with interstitial deletions of 22q13 that preserve *SHANK3.* To reduce the confusion for families, clinicians, researchers, and pharma, a simple classification for PMS has been devised. PMS and will be further classified as PMS-*SHANK3* related or PMS-*SHANK3* unrelated. PMS can still be used as a general term, but this classification system is inclusive. It allows researchers, regulatory agencies, and other stakeholders to define *SHANK3* alterations or interstitial deletions not affecting the *SHANK3* coding region.

The purpose of this article is to define a classification system for Phelan-McDermid syndrome (PMS) [OMIM #606232] that distinguishes between cases involving deletion or pathogenic variants of *SHANK3* and cases in which *SHANK3* is not disrupted. This system ensures that individuals previously diagnosed with PMS based on an interstitial deletion of 22q13 proximal to *SHANK3* will now meet the criteria for the syndrome, and that those with deletion of 22q13 and intact *SHANK3* whose clinician did not feel they fit the definition of PMS will now have a diagnosis.

The classification system is simple, straightforward, and ensures inclusion for all individuals who have a deletion of the distal long arm of chromosome 22. PMS will be classified as PMS-*SHANK3* related or PMS-*SHANK3* unrelated.

Historically, PMS was initially described as a 22q13 deletion, without knowledge of the existence or role of *SHANK3* [1–3]. Early reports described terminal deletions, interstitial deletions, unbalanced translocations, ring chromosomes, insertions, and recombinant chromosomes as leading to 22q13 deletion syndrome [4–9]. Later studies pointed to deletions, disruptions, or pathogenic variants of the *SHANK3* gene as the cause of the neurobehavioral phenotype in PMS [10–12]. Because some interstitial deletions are proximal to the *SHANK3* locus [13–16], confusion ensued as to whether individuals with these interstitial deletions of 22q13 should be diagnosed with PMS.

The *SHANK3* gene maps to 22q13.3, is expressed broadly in the brain and codes for a large scaffold protein within the post-synaptic density of neuronal excitatory synapses [17, 18]. Both the function and location of *SHANK3* make it a prime candidate for the neurological deficits in PMS, and genetic studies support this role. Bonaglia et al. [10] described the disruption of the *SHANK3* locus by a de novo balanced translocation, t(12;22)(q24.1;q13.3), in an individual with features of the 22q13 deletion syndrome. Two additional reports of disruption of *SHANK3* due to submicroscopic deletions (130 kb and 100 kb) and mild features of the syndrome have also been described [6, 19]. Investigations by Luciani et al. [11] and Wilson et al. [12] in their series of 32 and 56 patients, respectively, showed that *SHANK3* was in the minimum region of overlap responsible for the deletion 22q13 phenotype. Finally, the first cases of de novo* SHANK3* pathogenic variants were detected in patients with intellectual disability with or without autism, making *SHANK3* a major gene in understanding PMS [20–22]. *SHANK3* is therefore a significant factor increasing the probability of presenting autistic traits for patients with terminal 22q13 deletion, but as for other cases of autism, additional factors might still be required to have the full ASD diagnosis.

Because *SHANK3* is located at the terminal long arm of chromosome 22, most of the 22q13 deletions alter the coding region of *SHANK3*. This has led some researchers and clinicians to consider that *SHANK3* must be mutated (pathogenic variant or deleted) for a diagnosis of PMS. There are, however, reports of individuals with the PMS phenotype and interstitial deletions that do not disrupt *SHANK3* suggesting that other genes in the region may contribute to a similar phenotype and/or that a positional effect may influence *SHANK3* expression [23]. Wilson et al. [13] described 2 children with overlapping interstitial deletions who had intellectual impairment, delayed speech, hypotonia, abnormal MRI, and minor dysmorphic features. The parent of one child also had the deletion with mild speech impairment and normal cognition. Simenson et al. [15] reported an individual with a 720 kb interstitial deletion of 22q13.2, classic features of PMS and elevated immunoglobulin E. Nine individuals with overlapping interstitial deletions of 22q13 ranging from 2.7 to 6.9 Mb in size were reported by Disciglio et al. [14]. Although the deletions did not involve *SHANK3*, the patients had common, albeit non-specific, features of PMS, including developmental delay, speech delay, hypotonia, and feeding difficulties. The authors suggest that this is a new contiguous gene syndrome—distinct from PMS because *SHANK3* is not involved—and that *SULT4A1* and *PARVB* are candidate genes for the neurological features of this new syndrome. Interestingly, one feature that distinguishes many of the interstitial deletion patients from the “typical” PMS patient was macrocephaly [24]. However, Rollins et al. [25] examined head size in 53 patients with PMS and reported that the incidence of macrocephaly was significantly greater than expected (*p* < 0.001). Macrocephaly in PMS has also been associated with increased deletion size (median deletion size 6.99 Mb), with macrocephaly more likely associated with the larger interstitial deletions than the smaller deletions clustered around *SHANK3* [26].

The paucity of patients with interstitial deletions has led to a dearth of information about their clinical and intellectual profile when compared to the patients with terminal deletions or pathogenic variants of *SHANK3.* Further research is required on this group of individuals to determine how the guidelines for PMS are relevant to individuals who have deletions that do not alter *SHANK3.*

The phenotype of PMS is variable and non-specific. Neurobehavioral features include neonatal hypotonia, intellectual impairment, absent or delayed speech, and autism or autistic-like behavior. Physical features, such as long eyelashes, hypoplastic toenails, and fleshy hands, are more common in individuals with PMS than in the general population [27]. The diagnosis of PMS*-SHANK3* related is based on the detection of a deletion or disruption of 22q13 that affects the exons of *SHANK3* or on the demonstration of a pathogenic variant of *SHANK3.*

Deletions range in size from less than 1 kb to over 9 Mb. Patients with PMS-*SHANK3* unrelated must carry an interstitial deletion of 22q13 that does not affect the promoter or the exons of *SHANK3*. Patients should also present the main features commonly seen in PMS (see above). As mentioned before, at least 12 patients were reported in the literature with PMS-*SHANK3* unrelated (10 are present in the PMSF International Registry).

Most of the patients with 22q13 interstitial deletions not affecting *SHANK3* coding regions share an overlapping constellation of features similar to patients with terminal deletions including *SHANK3*, inferring that they have the same syndrome and providing little definitive evidence that they represent separate syndromes. The use of PMS for all 22q13 deletion and to designate whether the coding region of *SHANK3* is or is not involved will resolve the confusion that has plagued clinicians, researchers, pharma, and families when dealing with interstitial deletions of 22q13.

## Data Availability

Data sharing is not applicable to this article as no datasets were generated or analyses during the current study.

## Abbreviations

PMS: Phelan-McDermid syndrome
PMSF: Phelan-McDermid syndrome foundation

## References

[1] Phelan MC, Rogers RC, Stevenson RE (1988). A de novo terminal deletion of 22q. Am J Hum Genet.

[2] Phelan MC, Thomas GR, Saul RA, Rogers RC, Taylor HA, Wenger DA (1992). Cytogenetic, biochemical, and molecular analyses of a 22q13 deletion. Am J Med Genet.

[3] Nesslinger NJ, Gorski JL, Kurczynski TW, Shapira SK, Siegel-Bartelt J, Dumanski JP (1994). Clinical, cytogenetic, and molecular characterization of seven patients with deletions of chromosome 22q13.3. Am J Hum Genet.

[4] Watt JL, Olson IA, Johnston AW, Ross HS, Couzin DA, Stephen GS (1985). A familial pericentric inversion of chromosome 22 with a recombinant subject illustrating a ‘pure’ partial monosomy syndrome. J Med Genet.

[5] Romain DR, Goldsmith J, Cairney H, Columbano-Green LM, Smythe RH, Parfitt RG (1990). Partial monosomy for chromosome 22 in a patient with del(22)(pter- > q13.1::q13.33- > qter). J Med Genet.

[6] Flint J, Wilkie AOM, Buckle VJ, Winter RM, Holland AJ, McDermid HE (1995). The detection of subtelomeric chromosomal rearrangements in idiopathic mental retardation. Nat Genet.

[7] Smith DP, Floyd M, Burhan S (1996). Detection of a familial cryptic translocation by fluoroescent in situ hybridization. J Med Genet.

[8] Slavotinek A, Maher E, Gregory P, Rowlandson P, Huson SM (1997). The phenotypic effects of chromosome rearrangement involving bands 7q21.3 and 22q13.3. J Med Genet.

[9] Phelan MC, Rogers RC, Saul RA, Stapleton GA, Sweet K, McDermid HE (2001). Research review: 22q13 deletion syndrome. Am J Med Genet.

[10] Bonaglia MC, Giorda R, Borgatti R, Felisari G, Gagliardi C, Selicorni A (2001). Disruption of the ProSAP2 gene in a t (12;22)(q24.1;q13.3) associated with the 22q13.3 deletion syndrome. Am J Hum Genet.

[11] Luciani JJ, de Mas P, Depetris D, Mignon-Ravix C, Bottani A, Prieur M (2003). Telomeric 22q13 deletions resulting from rings, simple deletions, and translocations: cytogenetic, molecular, and clinical analyses of 32 new observations. J Med Genet.

[12] Wilson HL, Wong ACC, Shaw SR, Tse W-Y, Stapleton GA, Phelan MC (2003). Molecular characterisation of the 22q13 deletion syndrome supports the role of haploinsufficiency of *SHANK*3/*PROSAP*2 in the major neurological symptoms. J Med Genet.

[13] Wilson HL, Crolla JA, Walker D, Artifoni L, Dallapiccola B, Takano T (2008). Interstitial 22q13 deletions: genes other than SHANK3 have major effects on cognitive and language development. Eur J Hum Genet.

[14] Disciglio V, Rizzo CL, Mencarelli MA, Mucciola M, Marozza A, Di Marco C (2014). Interstitial 22q13 deletions not involving SHANK3 gene: a new contiguous gene syndrome. Am J Med Genet A.

[15] Simenson K, Oiglane-Shlie E, Teek R, Kuus K, Ounap K (2017). A patient with the classic features of Phelan-McDermid syndrome and a high immunoglobulin E level caused by a cryptic interstitial 0.72-Mb deletion in the 22q13.1 region. Am J Med Genet A.

[16] Phelan K, Boccuto L, Rogers RC, Sarasua SM, McDermid HE (2015). Letter to the editor regarding Disciglio et al.: interstitial 22q13 deletions not involving SHANK3: a new contiguous gene syndrome. Am J Med Genet A.

[17] Naisbitt S, Kim E, Tu JC, Xiao B, Sala C, Valtschanoff J (1999). Shank, a novel family of postsynaptic density proteins that binds to the NMDA receptor/PSD-95/GKAP complex and cortactin. Neuron.

[18] Boeckers TM, Bockmann J, Kreutz MR, Gundelfinger ED (2002). ProSAP/Shank proteins—a family of higher order organizing molecules of the postsynaptic density with an emerging role in human neurological disease. J Neurochem.

[19] Anderlid BM, Schoumans J, Annerén G, Tapia-Paez I, Dumanski J, Blennow E (2002). FISH-mapping of a 100-kb terminal 22q13 deletion. Hum Genet.

[20] Durand CM, Betancur C, Boeckers TM, Bockmann J, Chaste P, Fauchereau F (2007). Mutations in the gene encoding the synaptic scaffolding protein SHANK3 are associated with autism spectrum disorders. Nat Genet.

[21] Leblond CS, Nava C, Polge A, Gauthier J, Huguet G, Lumbroso S (2014). Meta-analysis of SHANK Mutations in Autism Spectrum Disorders: a gradient of severity in cognitive impairments. PLoS Genet.

[22] De Rubeis S, Siper PM, Durkin A, Weissman J, Muratet F, Halpern D (2018). Delineation of the genetic and clinical spectrum of Phelan-McDermid syndrome caused by SHANK3 point mutations. Mol Autism.

[23] Tabet AC, Rolland T, Ducloy M, Lévy J, Buratti J, Mathieu A (2017). A framework to identify contributing genes in patients with Phelan-McDermid syndrome. NPJ Genom Med.

[24] Mari F, Novelli A, Romano C, Alessandra R (2015). Response to Phelan K et al: Letter to the Editor Regarding Disciglio et al: interstitial 22q13 deletion not involving SHANK3 gene: a new contiguous gene syndrome. Am J Med Genet A.

[25] Rollins JD, Sarasua SM, Phelan K, DuPont BR, Rogers CR, Collins JS (2011). Growth in Phelan-McDermid syndrome. Am J Med Genet Part A.

[26] Sarasua SM, Dwiviedi A, Boccuto L, Rollins JD, Chen C-F, Rogers RC (2011). Association between deletion size and important phenotypes expands the genomic region of interest in Phelan-McDermid syndrome (22q13 deletion syndrome). J Med Genet.

[27] Sarasua SM, Boccuto L, Sharp JL, Dwivedi A, Chen C-F, Rollins JD (2014). Clinical and genomic evaluation of 201 patients with Phelan-McDermid syndrome. Hum Genet.

